# Chromosome microarray testing for patients with congenital heart defects reveals novel disease causing loci and high diagnostic yield

**DOI:** 10.1186/1471-2164-15-1127

**Published:** 2014-12-17

**Authors:** Juan Geng, Jonathan Picker, Zhaojing Zheng, Xiaoqing Zhang, Jian Wang, Fuki Hisama, David W Brown, Mary P Mullen, David Harris, Joan Stoler, Ann Seman, David T Miller, Qihua Fu, Amy E Roberts, Yiping Shen

**Affiliations:** Department of Laboratory Medicine, Shanghai Children’s Medical Center, Shanghai Jiaotong University School of Medicine, Shanghai, 200127 China; Department of Laboratory Medicine, Boston Children’s Hospital, Boston, MA 02115 USA; Division of Genetics, Boston Children’s Hospital, Boston, MA 02115 USA; Department of Cardiology, Boston Children’s Hospital, Boston, MA 02115 USA; Harvard Medical School, Boston Children’s Hospital, Boston, MA 02115 USA; Claritas Genomics, Cambridge, MA 02139 USA; Division of Medical Genetics, University of Washington, Seattle, USA

**Keywords:** Congenital heart defects, Chromosomal microarray analysis, Copy number variant, Diagnostic yield

## Abstract

**Background:**

Congenital heart defects (CHD), as the most common congenital anomaly, have been reported to be frequently associated with pathogenic copy number variants (CNVs). Currently, patients with CHD are routinely offered chromosomal microarray (CMA) testing, but the diagnostic yield of CMA on CHD patients has not been extensively evaluated based on a large patient cohort. In this study, we retrospectively assessed the detected CNVs in a total of 514 CHD cases (a 422-case clinical cohort from Boston Children's Hospital (BCH) and a 92-case research cohort from Shanghai Children’s Medical Center (SCMC)) and conducted a genotype-phenotype analysis. Furthermore, genes encompassed in pathogenic/likely pathogenic CNVs were prioritized by integrating several tools and public data sources for novel CHD candidate gene identification.

**Results:**

Based on the BCH cohort, the overall diagnostic yield of CMA testing for CHD patients was 12.8(pathogenic CNVs)-18.5% (pathogenic and likely pathogenic CNVs). The diagnostic yield of CMA for syndromic CHD was 14.1-20.6% (excluding aneuploidy cases), whereas the diagnostic yield for isolated CHD was 4.3-9.3%. Four recurrent genomic loci (4q terminal region, 15q11.2, 16p12.2 and Yp11.2) were more significantly enriched in cases than in controls. These regions are considered as novel CHD loci. We further identified 20 genes as the most likely novel CHD candidate genes through gene prioritization analysis.

**Conclusion:**

The high clinical diagnostic yield of CMA in this study provides supportive evidence for CMA as the first-line genetic diagnostic tool for CHD patients. The CNVs detected in our study suggest a number of CHD candidate genes that warrant further investigation.

**Electronic supplementary material:**

The online version of this article (doi:10.1186/1471-2164-15-1127) contains supplementary material, which is available to authorized users.

## Background

Chromosomal microarray (CMA) analysis, which can better define the size of microdeletions/microduplications and their gene content, enables novel disease gene discoveries and genotype-phenotype correlation studies [1, 2]. The diagnostic yield of CMA testing ranges from approximately 5% to 20% for patients with developmental delay/intellectual disability (DD/ID), autism spectrum disorder (ASD), or multiple congenital anomalies (MCAs), significantly higher than that of G-banded karyotyping (3%) [3]. The American College of Medical Genetics and Genomics (ACMG)recommends the use of CMA as thefirst-tier diagnostic test for these patients [4].

Congenital heart defect (CHD) is among the most common birth defects and is a leading cause of infant mortality around the world. It affects approximately 0.8-1% of newborns [5, 6]. Recent studies have shown that pathogenic CNVs are identified in a substantial proportion of CHD patients [7, 8]. Multiple recurrent CNV loci such as 22q11.2 (the DiGeorge syndrome region), 7q11.23, 8p23.1, 9q34.3, and 1q21.1 were found to confer significant risk for syndromic or isolated CHD [9–11]. These loci only explain a fraction of the genetic underpinnings of CHD [7]. In recent years, CMA has been routinely offered to patients with CHD. Several studies have evaluated and reported the clinical diagnostic yields of such practice but largely based on small patient cohorts [12–19]. Clinical diagnostic CMA data have proven to be an invaluable source for genetic discoveries and genotype-phenotype correlation studies. Here, we retrospectively reviewed the CNV detection in unselected clinical CHD cases at the Genetic Diagnostic Laboratory of Boston Children's Hospital (BCH) and selected research CHD cases from Shanghai Children’s Medical Center (SCMC). We assessed the clinical significance of each CNV and evaluated the overall diagnostic yield. We further uncovered novel CHD-associated CNVs and potential CHD candidate genes through gene prioritization and pathway analysis.

## Methods

### Study subjects and phenotype classification

422 patients (56% male and 44% female, median age = 7 years) with at least one congenital heart defect who underwent clinical CMA testing at BCH between December 2006 and April 2013 were included in this study. The relevant medical records, including clinical notes and echocardiography reports, were reviewed. In addition, 92 CHD patients (61 male and 31 female, median age = 3 years) from SCMC were included in this study. This group of patients was evaluated by echocardiography, magnetic resonance imaging, cardiac catheterization or surgical reports to determine the type of CHD. SCMC patients with gross chromosomal aberrations (e.g., trisomy 21 and trisomy 18) were excluded from CMA analysis. Both studies were approved by respective IRBs of Boston Children's Hospital and Shanghai Children’s Medical Center. Informed consent for patients from SCMC was obtained from parents. No identifiable information was used in the manuscript. Cases ascertained at BCH included all CHD phenotypes that were further subcategorized using the classification system established by National Birth Defects Prevention Study (NBDPS) [20], whereas cases ascertained at SCMC primarily had conotruncal defects (CTD). Patients who only had mild CHD abnormalities (i.e. isolated patent ductus arteriosus and patent foramen ovale) or were affected only by arrhythmia or cardiomyopathy were excluded from this study.

For comparison of CNV detection rate, a control cohort was assembled from previously published studies [8, 21, 22], which used high-density microarray platforms comparable to the ones used in this study.

### Chromosomal Microarray testing and CNV evaluation

DNA samples from all cases were extracted from peripheral blood with standard procedures. CHD patients at BCH were tested on the Agilent 244 K comparative genomic hybridization (CGH) array platform or a 4 × 180 K SNP + CGH microarray in a clinical diagnostic setting. CNVs were identified and evaluated as previously described [23].

CHD cases at SCMC were tested on the Affymetrix Cytoscan™ HD microarray platform in a research setting. Data was visualized and analyzed by Chromosome Analysis Suite (ChAS) software package (Affymetrix, USA) with a minimal cutoff of 20 consecutive markers for CNV calling. All CNVs reported are based on NCBI human genome build 37 (hg 19).

Detected CNVs were evaluated through a filtering procedure and classified into five categories based on the ACMG guideline [24] (for details see Additional file 1).

### Statistical analysis

Two-sided Fisher’s exact test was used to compare the frequencies of recurrent (n ≥ 3) CNVs between the case and the control cohorts, the CNV detection rates between isolated CHD and syndromic CHD, and the CNV burden for each subcategory of CHD. A p value < 0.05 was considered significant throughout this study.

### Gene prioritization for novel CHD candidate gene identification

We developed an analytic process by integrating various tools and data sources to prioritize the genes involved in detected CNVs. RefSeq genes encompassed in the pathogenic CNVs and likely pathogenic CNVs were assembled as the starting gene list. The genes in deletions and duplications were analyzed separately (Additional file 1). Independently, we also used the same prioritization process to evaluate the novel CHD candidate genes involved in the pathogenic CNV(s) of each patient.

## Results

### Diagnostic yield of CMA testing for patients with CHD

Among 422 CHD patients from BCH, 12 individuals were found to have gross chromosomal aberrations including five trisomy 21, five monosomy X, one trisomy 18, and one 18q partial trisomy. In the remaining patients, we detected 50 pathogenic CNVs in 42 patients (10.2%) and 28 likely pathogenic CNVs in 24 patients (5.8%) (Additional file 1: Table S1, S2). The overall diagnostic yield of CMA testing for patients with CHD was 18.5% when considering pathogenic, likely pathogenic CNVs and aneuploidies as positive finding. The minimal diagnostic yield was 12.8% if only the cases with pathogenic genomic imbalances (including aneuploidies) were included. The majority of pathogenic CNVs (~74%) detected in patients were smaller than 10 Mb in size, which would presumably be missed by karyotyping, again demonstrating the superior technical validity of microarray in detecting clinically relevant CNVs over karyotyping.

### Diagnostic yield of CMA in syndromic vs. non-syndromic CHD

All 12 patients carrying aneuploidy exhibited a syndromic CHD phenotype. The remaining 410 individuals were divided into two groups: isolated CHD or syndromic CHD based on medical records. The former consisted of 162 patients and the latter consisted of 248 individuals exhibiting extracardiac phenotypes in addition to heart defects. The most common extracardiac phenotypes were ID/DD, ASD, behavioral features, hypotonia and craniofacial dysmorphism. Even after excluding aneuploidy cases, there were significantly more non-polymorphic CNVs (CNVs not recurrent in general population) in syndromic CHD than in isolated CHD (Table 1, p = 0.0078). The p value for pathogenic CNVs only (p = 0.0013) and for pathogenic and likely pathogenic combined category (p = 0.0024) also reached statistical significance. Based on this analysis, the diagnostic yield of CMA for isolated CHD was 4.3% (pathogenic CNVs)-9.3% (pathogenic and likely pathogenic CNVs), whereas the diagnostic yield for all syndromic CHD (excluding aneuploidy cases) is 14.1(pathogenic CNVs)-20.6% (pathogenic and likely pathogenic CNVs) (Table 1).

**Table 1:** **Association of CNV with isolated CHD and syndromic CHD**

CNV category	Syndromic CHD cases ^***a***^				Isolated CHD cases (n = 162)	Syndromic CHD vs. Isolated CHD p Value
	Syndromic CHD cases with co-occurring DD/ID or ASD (n = 75)	Syndromic CHD cases without DD/ID and ASD(n = 173)	p Value	Total cases (n = 248)		
Non-polymorphic CNVs	38 (50.7%)	60 (34.7%)	**0.0234**	98 (39.5%)	43 (26.5%)	**0.0078**
Pathogenic CNVs	17 (22.7%)	18 (10.4%)	**0.0163**	35 (14.1%)	7 (4.3%)	**0.0013**
Pathogenic + likely pathogenic CNVs	22 (29.3%)	29 (16.7%)	**0.0275**	51 (20.6%)	15 (9.3%)	**0.0024**

^*a*^Twelve patients with aneuploidy were not included.

The following abbreviations were used: DD, development delay; ID, intellectual disability; ASD, autism spectrum disorder.

### Diagnostic yield of CMA related to CHD sub-types

We further compared the CNV detection rates between syndromic CHD with co-occurring neurodevelopmental disorders (NDD) including DD, ID and ASD with those without NDD. Twice as many pathogenic CNVs were detected in CHD patients with NDD than those without NDD (Table 1), indicating that patients with co-morbid features of CHD and NDD were more likely to harbor pathogenic CNVs. This finding also suggested that CNVs detected in syndromic CHD patients were not solely contributing to NDD which are known to be associated with CNVs.

To further delineate the association of CHD sub-categories with CMA detection rates, we classified the BCH cases into nine categories (Additional file 1: Table S3). Among patients with isolated CHD, those with compound CTD (category F), hypoplastic left heart syndrome (category G) and obstruction of left ventricular outflow tract (category D) were more likely to harbor pathogenic CNVs (Table 2). When all CHD cases were considered, patients with isolated CTD (category E) exhibited the highest CMA diagnostic rate (14.8%). In addition, CHD patients with compound CTD (category F) and septal defects (category A) also reached a >10% diagnostic rate. In contrast, CHD patients with heterotaxy (category H) or valve defects (categories C) were less likely to have a pathogenic CNV.

**Table 2:** **Clinical relevance of CNV to CHD phenotypes**

	A	C	D	E	F	G	H
**Isolated CHD cases**							
No. of cases	n = 13	n = 19	n = 26	n = 8	n = 44	n = 31	-
Non-polymorphic CNV	4 (30.8%)	1 (5.3%)	7 (26.9%)	1(12.5%)	17 (38.6%)	7 (22.6%)	-
Pathogenic CNV	0	0	1 (3.9%)	0	3 (6.8%)	2 (6.5%)	-
Pathogenic + likely pathogenic CNV	0	0	4 (15.4%)	0	5 (11.4%)	3 (9.7%)	-
**All CHD cases**							
No. of cases	n = 84	n = 41	n = 74	n = 27	n = 75	n = 47	n = 23
Non-polymorphic CNV	34(40.5%)	6(14.6%)	25(33.8%)	12(44.4%)	31(44.3%)	14(29.8%)	4(17.4%)
Pathogenic CNV	11(13.1%)	2 (4.9%)	6 (8.1%)	4 (14.8%)	10(13.3%)	3(6.4%)	1(4.4%)
Pathogenic + likely pathogenic CNV	17(20.2%)	2 (4.9%)	13(17.6%)	4 (14.8%)	13(17.3%)	5(10.6%)	3(13.0%)

The cases of B category was too low, thus not included. The twelve patients with aneuploidy were not included for calculation.

Among 92 SCMC patients mainly affected with CTD, a total of 26 non-polymorphic CNVs were detected, 11 of them were classified as pathogenic or likely pathogenic (Additional file 1: Table S1, S2). The CNV detection rate for this cohort was about 12%, which is similar to that of BCH patients with the same sub-phenotype, thus we independently confirmed the significant involvement of CNV in CTD.

### Identification of known and novel recurrent CNVs associated with CHD

**Figure 1:**
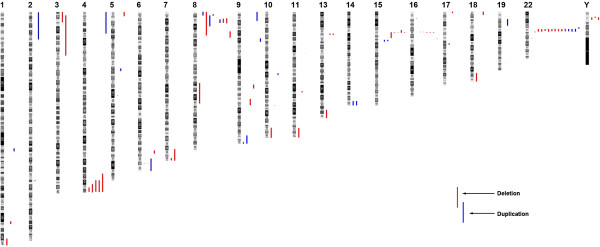
**Distribution of the 89 sub-chromosomal genomic imbalances detected in this study among patients with CHD.** Chromosomal loci 22q11.21 and 8p23.1 were two known pathogenic CNV hotspots in CHD patients. This study also identified deletions at loci 4q terminal, 15q11.2, 16p12.1 and Yp11.2 as potential pathogenic hotspots.

**Figure 2:**
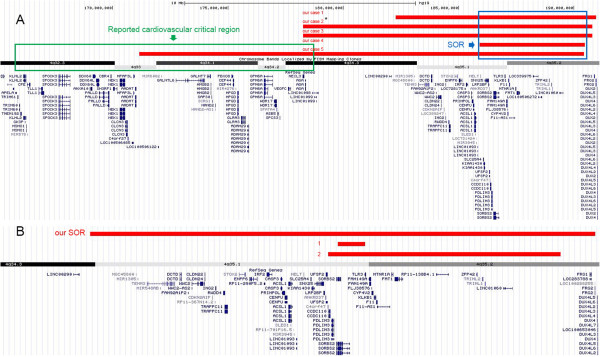
**Recurrent deletions in the 4q terminal region. (A)** The previously reported cardiovascular critical region (4q32.2-q34.3) are located proximal to the smallest overlapping region (SOR) defined in this study. The two regions do not overlap. The asterisk indicates the *de novo* variant. **(B)** Two additional cases with deletion overlap with the SOR. Cases 1 from Strehle, EM, et al. [30] and Case 2 from Maurin et al. [29]. All three 4q terminal deletions involved the *SORBS2* gene.

A genome-wide CNV analysis for a total of 502 CHD cases (410 from BCH cohort and 92 from SCMC cohort; Additional file 1: Figure S1) led to the detection of 209 (183 in BCH cohort and 26 in SCMC cohort) non-polymorphic CNVs. As a result, a total of 89 CNVs at 57 unique chromosome loci were considered to be of known or possible clinical relevance in this study. They were widely distributed on different chromosomes (Figure 1). We observed 32 recurrent (n ≥ 3) CNVs distributed at six chromosomal loci (Additional file 1: Table S1, S2) which include 12 imbalances (nine deletions and three duplications) at 22q11.2 and five aberrations (three deletions and two duplications) at the 8p23.1 involving the *GATA4* gene, both loci are known to be associated with syndromic or isolated CHD. In this study, we also identified five patients with 4q terminal deletions which range from 4, 600 kb to 19, 300 kb in size (Figure 2A). Similar deletions were not detected in 9170 control cases (Table 3), and are not reported in DGV (http://dgv.tcag.ca/ accessed March, 2014). 4q terminal deletion is known to cause 4q- syndrome where 50% of affected individuals have CHD, and a cardiovascular critical region has been narrowed down to 4q32.2–q34.3 [25]. The smallest overlapping region (SOR) among our 4q terminal deletion cases was about 4.6 Mb in size at 4q35.1-qter. This SOR didn’t overlap with the previously defined critical region (Figure 2A). Thus our study potentially maps a novel CHD critical region at the 4q terminus. There were 24 Refseq genes at this interval, although no known CHD genes existed, we propose several possible candidate genes in discussion.

**Table 3:** **Recurrent CHD-associated CNV loci**

Locus (hg19)	CNV	Size range (kb)	Cardiac Phenotypes	Frequency in our study	Frequency in control	p Value
4qter	Deletion	4559-19269	DORV, right dominant AV canal, hypoplastic LV, multiple VSDs, BAV, CoA, DILV, hypoplastic aortic arch, TOF, PFO	5/502	0/9170^*a*^	**<0.0001**
15q11.2	Deletion	245-2703	D-TGA, VSD, ASD, PS, CoA, PDA, AS, AR, left ventricular dysfunction	4/502	19/9170	**0.0289**
16p12.2^*c*^	Deletion	480	ASD, TAPVC, PDA	3/502	3/9170	**0.0025**
Yp11.2	Deletion	1300-3000	ASD, CoA, PFO	3/502	12/32850^*b*^	**<0.0001**

^*a*^control cases from Soemedi et al. [8] and Cooper et al. [21].

^*b*^control cases from Repnikova et al. [22].

^*c*^The chromosome coordinates for this deletion map to 16p12.1 in hg18, but map to 16p12.2 in hg19.

In addition, we identified three other genomic loci with significantly higher frequencies in cases than in controls. These three loci were 15q11.2 (p = 0.0289), 16p12.2 (p = 0.0025) and Yp11.2 (p < 0.0001) (Table 3) respectively, which were also considered as possible novel loci associated with cardiac development.

### Identification of novel CHD candidate genes

Among 57 CNV regions of interest, ten CNVs contained genes known to be causal for CHD (Additional file 1: Figure S1 and Table S4). In order to identify novel CHD candidate genes, we examined the genes within the remaining 47 loci (Additional file 1: Table S5). Starting from 647 genes in deletion CNVs and 517 genes in duplication CNVs (Additional file 1: Figure S2), we performed a gene prioritization process using Endeavour and ToppGene. 18 genes in deletion CNVs and 18 genes (Additional file 1: Table S6 and Figure S2) in duplication CNVs in the category of "Cardiovascular System Development and Function" were identified as novel CHD candidate genes through mouse embryonic expression pattern analysis and Ingenuity Pathway Analysis (IPA) analysis (for details see Additional file 1).

Furthermore, the same gene prioritization process was performed for individual cases carrying pathogenic CNVs of unknown CHD significance. A total of 39 genes were identified in 19 cases (Additional file 1: Table S7). Of note, 20 of these genes were also contained in the global prioritization list (bold genes in Additional file 1: Table S7). These shared genes are considered to be the most likely dosage sensitive novel CHD candidate genes.

## Discussion

### Diagnostic yields of CMA testing

CMA has been recommended as the first-line test in the initial postnatal genetic evaluation of individuals with MCAs, DD/ID and ASD [4]. CHD is known to be frequently associated with CNVs (Additional file 1: Table S8). Currently, patients with CHD are routinely offered CMA testing. In contrast to previous publications (Table 4), the present study documents the diagnostic yields for several sub-categories of syndromic and non-syndromic CHD using the largest cohort.

**Table 4:** **Summary of the diagnostic yields of CMA in clinical setting with different microarray platforms in studies of CHD from literatures**

	Study	Platform	Patients		Diagnostic yield (%)
			No.	Phenotype	
1	Thienpont B, et al. (2007) [12]	1 Mb BAC/PAC	60	Syndromic CHD	10(16.6%)
2	Richards AA, et al. (2008) [13]	Nimblegen 385 K CGH	20	Syndromic CHD	5 (25%)
			20	Isolated CHD	0
3	Erdogan F, et al. (2008) [14]	tiling path BAC array	105	Isolated CHD	4(3.8%)
4	Breckpot J, et al. (2010) [15]	1 Mb BAC/PAC	90	Syndromic CHD	16(17.8%)
5	Goldmuntz E, et al. (2011) [16]	Affymetrix GeneChip 100 K array	58	Syndromic CHD	12(20.7%)
6	Breckpot J, et al. (2011) [17]	Affymetrix 6.0 array	46	Isolated CHD	2 (4.3%)
7	Connor JA, et al. (2013) [18]	Not specified	121	Unselected CHD	9 (7%)
8	Syrmou A, et al. (2013) [19]	Agilent 244 K CGH array	55	Syndromic CHD	29 (52.7%)^*a*^
		Agilent 4 × 180 K SNP + CGH array			
9	Our study	Same as above	162	Isolated CHD	7 (4.3%)
			260^*b*^	Syndromic CHD	47 (18.1%)

Diagnostic yield was defined as the number of patients with abnormal aberrations divided by the total number of cases tested. In patients with syndromic CHD, pathogenic chromosomal imbalances were detected in about 16%-25% of cases. But the diagnostic yield of CMA in isolated CHD cohort was poorly studied.

^*a*^Many CNVs included were not necessary pathogenic.

^*b*^Twelve patients with gross chromosomal aberrations were included.

Previous clinical studies have demonstrated a higher CNV diagnostic yield in syndromic CHD than that in isolated CHD, but these studies were all done separately for either syndromic CHD or isolated CHD by different array platforms (Table 4). Using the largest cohort of CHD from one clinical setting, we were able to assess the CMA diagnostic yields for both syndromic and isolated CHD patients by the same CMA platform. Our study convincingly demonstrated a significantly higher CNV detection rate in syndromic CHD (18.1%) than isolated CHD (4.3%).

Importantly, our data also revealed that the CNV diagnostic yields differ among different CHD subcategories, indicating different CHD sub-phenotypes may have different pathogenic mechanisms. The findings that isolated CTD, compound CTD, and septal defects were more likely to be associated with CNVs than heterotaxy or valve defects provided practical guideline for referring CHD patients for CMA testing. However, the number of cases in each sub-category was still small in this study. Research involving a larger sample size is warranted to further delineate the correlation between CNV rate and CHD sub-phenotypes.

In the clinical setting, the pathogenicity of a CNV is assessed based on gene content, CNV size, and literatures. In many instances, the causal relationship between a particular CNV and a particular phenotype cannot be easily established. It is likely that not all of the pathogenic CNVs detected are directly causative of CHD. Thus, the exact diagnostic yield of causal CNVs for CHD may be less than the overall pathogenic CNV detection rate (12.8%). We identified 19 CNVs with known CHD genes (Additional file 1: Table S2). In addition, we also detected 12 aneuploidies that are known to be causally associated with CHD phenotypes. Therefore, a total of 31 cases out of 422 (7.3%) have chromosomal imbalances that are known to cause CHD. Additionally, we believe that a significant fraction of the remaining CNVs currently with unproven causal relationship with CHD may turn out to be novel CHD loci (such as 4q terminal deletion and other novel candidate CHD loci).Thus, although the exact diagnostic yield of causative CNVs is difficult to assess, it is reasonable to believe that the actual diagnostic yields is higher than 7.3%, and somewhat smaller than 12.8%. This level of diagnostic yield is similar to that for ASD (7%) [26], DD/ID or MCAs (10-12%) [3, 27]. Thus our findings provide strong evidence for CMA to be used as the first-line genetic diagnostic test for patients with CHD as well.

Many CHD patients in our study exhibited comorbid features of DD, MCAs or ASD, which are known to have a significant association with CNV. The fact that syndromic CHD patients have a higher pathogenic CNV detection rate than cases with only DD/ID/ASD suggests that not all CNVs detected in our syndromic CHD patients can be attributed to the DD/ID or ASD phenotype. Our data demonstrated an additive effect on CNV burden when these phenotypes co-occur with CHD. The fact that patients with isolated CHD exhibited a CNV diagnostic rate of 4.3% further supports the significant contribution of CNV to the pathogenesis of CHD.

High diagnostic yields provided strong supporting evidence to justify the routine use of CMA test in clinical evaluation of patients with CHD. While diagnostic yield is important, we are also interested to assess the clinical utility of CMA test for patients with CHD. A follow-up study will be focusing on how the CMA test results impact patient care and management.

### Discovering novel CHD loci and candidate genes through CNV detection

In this study, the top five most frequently detected genomic imbalance events in CHD cases were 22q11.2 deletion/duplication, 8p23.1 deletion/duplication, trisomy 21, monosomy X and 4q terminal deletion. The first four genomic imbalances are known to be causally related to CHD. *TBX1* and *GATA4* are the known key causative genes for CHD phenotypes for 22q11.2 and 8p23.1, respectively. The fact that all five 4q terminal deletion cases were detected in CHD patients and none in the control strongly supports the notion that 4q terminal deletion is a novel CHD-causing locus.

4q terminal deletion is a subgroup of 4q- syndrome, which has CHD in about 50% of the cases. Our five patients presented different CHD phenotypes, including CTD, hypoplastic left ventricle, septal defects and obstruction of left ventricular outflow tract. Additionally, three of them have comorbid features of extracardiac presentations (Additional file 1). A cardiovascular critical region (4q32.2-q34.3) has been mapped for the 4q- syndrome, and three genes (*TLL1, HPGD, and HAND2*) were proposed to be the key genes responsible for the cardiovascular phenotypes [25]. Interestingly, the SOR region of our five 4q terminal deletion cases does not overlap with this cardiovascular critical region (Figure 2A), suggesting that our 4.6 Mb region represents a novel CHD critical locus.

4q terminal deletions often co-occur with terminal duplications of other chromosome as a consequence of imbalanced segregation of a balanced parental translocation. In our study, the three largest 4q terminal deletion cases also carried terminal duplications on another chromosome (Additional file 1). The fact that the other involved chromosomes were different in each case and that the remaining two cases only carried the pathogenic 4q terminal deletion makes a strong argument that it is the genes within the 4q terminal deletion region, not on the other involved chromosomes, that are causal for CHD phenotype. Through literature review, we identified two other CHD cases with small 4q terminal deletions that overlapped with our SOR region (Figure 2B). All three 4q terminal deletions involved the *SORBS2* gene, encoding a signal transducer that is highly and nearly exclusively expressed in epithelia and cardiac muscle tissue in the mouse embryo (Additional file 1: Figure S3A). Strong expression in cardiac tissue suggests that this gene may play a significant role in heart development. Several previous studies also support the *SORBS2* gene as a critical gene for CHD [28–30].Our SOR and case 2 (Figure 2B) also contained the *PDLIM3* gene. The functional disruption of *Pdlim3* in mice results in right ventricular dysmorphogenesis, trabeculation failure, and chamber dilatation [31, 32], supporting the involvement of this gene in heart development. Maurin et al. suggested both *PDLIM3* and *SORBS2* were involved in cardiac and muscle development, and could be responsible for cardiac defects observed in terminal 4q35.1 deletions [29]. Additionally, the *SLC25A4* gene (MIM 103220), which encodes a member of the mitochondrial carrier subfamily of solute carrier protein, was previously associated with familial hypertrophic cardiomyopathy [33] and showed high expression in mouse embryonic heart (Additional file 1: Figure S3B). In fact, one case with 4q terminal deletion in our study presented with cardiomyopathy. It is currently unknown if any single gene at 4q terminal is sufficient to cause CHD, or if CHD occurs due to multiple gene deletion. Based on the above analysis, we propose that *SORBS2, PDLIM3* and *SLC25A4* are the critical genes associated with 4q terminal deletion for the CHD phenotype.

Recurrent deletions at locus 15q11.2 were statistically enriched in our CHD cohort. The region between BP1 and BP2 at 15q11.2 has been previously implicated as a contributory genetic cause of susceptibility to schizophrenia, behavioral disturbances, and intellectual disability [34, 35]. It is well known that the 15q11.2 deletion has low penetrance (for example only 2% for schizophrenia) [36]. Soemedi et al. was the first one to report the strong association of this variant with the risk of multiple heart defects, especially left-sided malformations [8]. However, no additional study followed. Our study provides independent support for the contributory role of 15q11.2 in CHD pathogenesis. We detected a total of 33 cases with 15q11.2 deletion. Three of them (9.1%) exhibited CHD phenotypes. Thus the penetrance of 15q11.2 deletion for CHD is also low. Additional genetic factors may be required for the manifestation of CHD.

We also identified three 16p12.1(hg 18) microdeletions involving the *EEF2K* and *CDR2* genes, which have been previously linked to intellectual disability and neuropsychiatric phenotypes [37]. Other features including cardiac anomalies are frequently observed in individuals with 16p12.1 deletion. Girirajan et al. identified seven individuals with CHD phenotype out of 21cases carrying this imbalance [38], suggesting its significant predisposing role to heart malformations. In our study, three out of five patients with 16p12.1 deletion exhibited CHD phenotype, demonstrating a relative high CHD penetrance of this imbalance.

CNVs detected in CHD patients provide a unique source for identifying novel CHD candidate genes. In this study, using gene prioritization approaches, we identified 20 novel candidate genes (11 genes in deletion CNVs and nine genes in duplication CNVs, Additional file 1: Table S9). We gathered additional supporting evidence including gene expression and mouse phenotype (Additional file 1: Table S9). We found that all of them had positive expression in mouse embryonic or adult heart. Some genes such as *Ets1, Nfatc1, Cnn1* and *Rps6ka2* exhibited a high expression level. The knock-out mice of all genes in deletion CNVs (except *Ptch1)* exhibited abnormal cardiovascular development. Of note, mice homozygotes for the targeted null allele of *Crk, Efnb2, Hey1, Nfatc1* and *Shh* display defects in heart morphogenesis. Although the function of these genes on human heart development is still poorly studied, two heterozygous mutations in *NFATC1* were recently reported in a patient with tricuspid atresia [39], and another recent study supported that *NFATC1* plays an important role in cardiac development [40]. For genes in duplication CNVs, *Dll1* knock-in mice and mice with mutations of the *Qki* gene displayed CHD involving impaired blood vessel morphology and abnormal heart looping. Taken together, these 20 genes are considered to be the most likely candidate CHD genes. Mutation screening in human CHD patients and functional studies will provide further evidence to demonstrate their causal relevance with CHD.

## Conclusion

In summary, the high clinical diagnostic yield of CMA for patients with CHD justify CMA to be used as a first-tier genetic test. Syndromic CHD cases are expected to have a much higher pathogenic CNV detection rate. CMA also provides diagnostic value for isolated CHD patients. The CNVs detected in CHD patients represent a wealth of CHD candidate genes that warrant further investigation.

## Electronic supplementary material

Additional file 1: **Supplementary data.** (DOCX 4 MB)

## Abbreviations

CHD: Congenital heart disease
CNV: Copy number variant
CMA: Chromosomal microarray assay
BCH: Boston Children’s Hospital
SCMC: Shanghai Children’s Medical Center
DD: Development delay
ID: Intellectual disorder
ASD: Autism spectrum disorder
MCAs: Multiple congenital anomalies
ACMG: American College of Medical Genetics and Genomics
NBDPS: National Birth Defects Prevention Study
CTD: Conotruncal defects
CGH: Comparative genomic hybridization
VOUS: Variation of uncertain significance
NDD: Neurodevelopmental disorders
SOR: The smallest overlapping region
HLHS: Hypoplastic Left Heart Syndrome.

## References

[1] Kaminsky EB, Kaul V, Paschall J, Church DM, Bunke B, Kunig D, Moreno-De-Luca D, Moreno-De-Luca A, Mulle JG, Warren ST, Richard G, Compton JG, Fuller AE, Gliem TJ, Huang S, Collinson MN, Beal SJ, Ackley T, Pickering DL, Golden DM, Aston E, Whitby H, Shetty S, Rossi MR, Rudd MK, South ST, Brothman AR, Sanger WG, Iyer RK, Crolla JA (2011). An evidence-based approach to establish the functional and clinical significance of copy number variants in intellectual and developmental disabilities. Genet Med.

[2] Riggs ER, Jackson L, Miller DT, Van Vooren S (2012). Phenotypic information in genomic variant databases enhances clinical care and research: The international standards for cytogenomic arrays consortium experience. Hum Mutat.

[3] Miller DT, Adam MP, Aradhya S, Biesecker LG, Brothman AR, Carter NP, Church DM, Crolla JA, Eichler EE, Epstein CJ, Faucett WA, Feuk L, Friedman JM, Hamosh A, Jackson L, Kaminsky EB, Kok K, Krantz ID, Kuhn RM, Lee C, Ostell JM, Rosenberg C, Scherer SW, Spinner NB, Stavropoulos DJ, Tepperberg JH, Thorland EC, Vermeesch JR, Waggoner DJ, Watson MS (2010). Consensus statement: chromosomal microarray is a first-tier clinical diagnostic test for individuals with developmental disabilities or congenital anomalies. Am J Hum Genet.

[4] Manning M, Hudgins L (2010). Array-based technology and recommendations for utilization in medical genetics practice for detection of chromosomal abnormalities. Gen Med.

[5] van der Linde D, Konings EEM, Slager MA, Witsenburg M, Helbing WA, Takkenberg JJM, Roos-Hesselink JW (2011). Birth prevalence of congenital heart disease worldwide. J Am Coll Cardiol.

[6] Reller MD, Strickland MJ, Riehle-Colarusso T, Mahle WT, Correa A (2008). Prevalence of congenital heart defects in Metropolitan Atlanta, 1998–2005. J Pediatr.

[7] Fahed A, Gelb B, Seidman J, Seidman C (2013). Genetics of congenital heart disease: the glass half empty. Circ Res.

[8] Soemedi R, Wilson Ian J, Bentham J, Darlay R, Töpf A, Zelenika D, Cosgrove C, Setchfield K, Thornborough C, Granados-Riveron J, Blue GM, Breckpot J, Hellens S, Zwolinkski S, Glen E, Mamasoula C, Rahman TJ, Hall D, Rauch A, Devriendt K, Gewillig M, O' Sullivan J, Winlaw DS, Bu'Lock F, Brook JD, Bhattacharya S, Lathrop M, Santibanez-Koref M, Cordell HJ, Goodship JA (2012). Contribution of global rare copy-number variants to the risk of sporadic congenital heart disease. Am J Hum Genet.

[9] Pierpont ME, Basson CT, Benson DW, Gelb BD, Giglia TM, Goldmuntz E, McGee G, Sable CA, Srivastava D, Webb CL (2007). Genetic basis for congenital heart defects: current knowledge: a scientific statement from the American heart association congenital cardiac defects committee, council on cardiovascular disease in the young: endorsed by the american academy of pediatrics. Circulation.

[10] Soemedi R, Topf A, Wilson IJ, Darlay R, Rahman T, Glen E, Hall D, Huang N, Bentham J, Bhattacharya S, Cosgrove C, Brook JD, Granados-Riveron J, Setchfield K, Bu'lock F, Thornborough C, Devriendt K, Breckpot J, Hofbeck M, Lathrop M, Rauch A, Blue GM, Winlaw DS, Hurles M, Santibanez-Koref M, Cordell HJ, Goodship JA, Keavney BD (2011). Phenotype-specific effect of chromosome 1q21.1 rearrangements and GJA5 duplications in 2436 congenital heart disease patients and 6760 controls. Hum Mol Genet.

[11] Greenway SC, Pereira AC, Lin JC, DePalma SR, Israel SJ, Mesquita SM, Ergul E, Conta JH, Korn JM, McCarroll SA, Gorham JM, Gabriel S, Altshuler DM, Quintanilla-Dieck Mde L, Artunduaga MA, Eavey RD, Plenge RM, Shadick NA, Weinblatt ME, De Jager PL, Hafler DA, Breitbart RE, Seidman JG, Seidman CE (2009). De novo copy number variants identify new genes and loci in isolated sporadic tetralogy of Fallot. Nat Genet.

[12] Thienpont B, Mertens L, de Ravel T, Eyskens B, Boshoff D, Maas N, Fryns J, Gewillig M, Vermeesch J, Devriendt K (2007). Submicroscopic chromosomal imbalances detected by array-CGH are a frequent cause of congenital heart defects in selected patients. Eur Heart J.

[13] Richards A, Santos L, Nichols H, Crider B, Elder F, Hauser N, Zinn A, Garg V (2008). Cryptic chromosomal abnormalities identified in children with congenital heart disease. Pediatr Res.

[14] Erdogan F, Larsen L, Zhang L, Tumer Z, Tommerup N, Chen W, Jacobsen J, Schubert M, Jurkatis J, Tzschach A, Ropers HH, Ullmann R (2008). High frequency of submicroscopic genomic aberrations detected by tiling path array comparative genome hybridisation in patients with isolated congenital heart disease. J Med Genet.

[15] Breckpot J, Thienpont B, Peeters H, de Ravel T, Singer A, Rayyan M, Allegaert K, Vanhole C, Eyskens B, Vermeesch JR, Gewillig M, Devriendt K (2010). Array comparative genomic hybridization as a diagnostic tool for syndromic heart defects. J Pediatr.

[16] Goldmuntz E, Paluru P, Glessner J, Hakonarson H, Biegel J, White P, Gai X, Shaikh T (2011). Microdeletions and microduplications in patients with congenital heart disease and multiple congenital anomalies. Congenit Heart Dis.

[17] Breckpot J, Thienpont B, Arens Y, Tranchevent LC, Vermeesch JR, Moreau Y, Gewillig M, Devriendt K (2011). Challenges of interpreting copy number variation in syndromic and non-syndromic congenital heart defects. Cytogenet Genome Res.

[18] Connor J, Hinton R, Miller E, Sund K, Ruschman J, Ware SM (2013). Genetic testing practices in infants with congenital heart disease. Congenit Heart Dis.

[19] Syrmou A, Tzetis M, Fryssira H, Kosma K, Oikonomakis V, Giannikou K, Makrythanasis P, Kitsiou-Tzeli S, Kanavakis E (2013). Array comparative genomic hybridization as a clinical diagnostic tool in syndromic and nonsyndromic congenital heart disease. Pediatr Res.

[20] Rasmussen SA, Olney RS, Holmes LB, Lin AE, Keppler-Noreuil KM, Moore CA (2003). Guidelines for case classification for the national birth defects prevention study. Birth Defects Res Part A Clin Mol Teratol.

[21] Cooper GM, Coe BP, Girirajan S, Rosenfeld JA, Vu TH, Baker C, Williams C, Stalker H, Hamid R, Hannig V, Abdel-Hamid H, Bader P, McCracken E, Niyazov D, Leppig K, Thiese H, Hummel M, Alexander N, Gorski J, Kussmann J, Shashi V, Johnson K, Rehder C, Ballif BC, Shaffer LG, Eichler EE (2011). A copy number variation morbidity map of developmental delay. Nat Genet.

[22] Repnikova EA, Rosenfeld JA, Bailes A, Weber C, Erdman L, McKinney A, Ramsey S, Hashimoto S, Lamb Thrush D, Astbury C, Reshmi SC, Shaffer LG, Gastier-Foster JM, Pyatt RE (2013). Characterization of copy number variation in genomic regions containing STR loci using array comparative genomic hybridization. Forensic Sci Int.

[23] Miller DT, Shen Y, Wu B-L (2012). Oligonucleotide microarrays for clinical diagnosis of copy number variation and zygosity status. Curr Protoc Hum Genet.

[24] Kearney HM, Thorland EC, Brown KK, Quintero-Rivera F, South ST (2011). American College of Medical Genetics standards and guidelines for interpretation and reporting of postnatal constitutional copy number variants. Gen Med.

[25] Xu W, Ahmad A, Dagenais S, Iyer RK, Innis JW (2012). Chromosome 4q deletion syndrome: narrowing the cardiovascular critical region to 4q32.2-q34.3. Am J Med Genet A.

[26] Shen Y, Dies KA, Holm IA, Bridgemohan C, Sobeih MM, Caronna EB, Miller KJ, Frazier JA, Silverstein I, Picker J, Weissman L, Raffalli P, Jeste S, Demmer LA, Peters HK, Brewster SJ, Kowalczyk SJ, Rosen-Sheidley B, McGowan C, Duda AW, Lincoln SA, Lowe KR, Schonwald A, Robbins M, Hisama F, Wolff R, Becker R, Nasir R, Urion DK, Milunsky JM (2010). Clinical genetic testing for patients with autism spectrum disorders. Pediatrics.

[27] Sagoo GS, Butterworth AS, Sanderson S, Shaw-Smith C, Higgins JPT, Burton H (2009). Array CGH in patients with learning disability (mental retardation) and congenital anomalies: updated systematic review and meta-analysis of 19 studies and 13,926 subjects. Gen Med.

[28] Rossi MR, DiMaio MS, Xiang B, Lu K, Kaymakcalan H, Seashore M, Mahoney MJ, Li P (2009). Clinical and genomic characterization of distal duplications and deletions of chromosome 4q: Study of two cases and review of the literature. Am J Med Genet A.

[29] Maurin ML, Labrune P, Brisset S, Le Lorc'h M, Pineau D, Castel C, Romana S, Tachdjian G (2009). Molecular cytogenetic characterization of a 4p15.1-pter duplication and a 4q35.1-qter deletion in a recombinant of chromosome 4 pericentric inversion. Am J Med Genet A.

[30] Strehle E-M, Yu L, Rosenfeld JA, Donkervoort S, Zhou Y, Chen T-J, Martinez JE, Fan Y-S, Barbouth D, Zhu H, Vaglio A, Smith R, Stevens CA, Curry CJ, Ladda RL, Fan ZJ, Fox JE, Martin JA, Abdel-Hamid HZ, McCracken EA, McGillivray BC, Masser-Frye D, Huang T (2012). Genotype-phenotype analysis of 4q deletion syndrome: Proposal of a critical region. Am J Med Genet A.

[31] Pashmforoush M, Pomiès P, Peterson KL, Kubalak S, Ross J, Hefti A, Aebi U, Beckerle MC, Chien KR (2001). Adult mice deficient in actininassociated LIM-domain protein reveal a developmental pathway for right ventricular cardiomyopathy. Nat Med.

[32] Pomies P, Pashmforoush M, Vegezzi C, Chien KR, Auffray C, Beckerle MC (2007). The Cytoskeleton-associated PDZ-LIM Protein, ALP, Acts on Serum Response Factor Activity to Regulate Muscle Differentiation. Mol Biol Cell.

[33] Strauss KA, DuBiner L, Simon M, Zaragoza M, Sengupta PP, Li P, Narula N, Dreike S, Platt J, Procaccio V, Ortiz-González XR, Puffenberger EG, Kelley RI, Morton DH, Narula J, Wallace DC (2013). Severity of cardiomyopathy associated with adenine nucleotide translocator-1 deficiency correlates with mtDNA haplogroup. Proc Natl Acad Sci.

[34] Burnside RD, Pasion R, Mikhail FM, Carroll AJ, Robin NH, Youngs EL, Gadi IK, Keitges E, Jaswaney VL, Papenhausen PR, Potluri VR, Risheg H, Rush B, Smith JL, Schwartz S, Tepperberg JH, Butler MG (2011). Microdeletion/microduplication of proximal 15q11.2 between BP1 and BP2: a susceptibility region for neurological dysfunction including developmental and language delay. Hum Genet.

[35] Stefansson H, Rujescu D, Cichon S, Pietiläinen OPH, Ingason A, Steinberg S, Fossdal R, Sigurdsson E, Sigmundsson T, Buizer-Voskamp JE, Hansen T, Jakobsen KD, Muglia P, Francks C, Matthews PM, Gylfason A, Halldorsson BV, Gudbjartsson D, Thorgeirsson TE, Sigurdsson A, Jonasdottir A, Jonasdottir A, Bjornsson A, Mattiasdottir S, Blondal T, Haraldsson M, Magnusdottir BB, Giegling I, Möller HJ, Hartmann A (2008). Large recurrent microdeletions associated with schizophrenia. Nature.

[36] Vassos E, Collier DA, Holden S, Patch C, Rujescu D, St Clair D, Lewis CM (2010). Penetrance for copy number variants associated with schizophrenia. Hum Mol Genet.

[37] Antonacci F, Kidd JM, Marques-Bonet T, Teague B, Ventura M, Girirajan S, Alkan C, Campbell CD, Vives L, Malig M, Rosenfeld JA, Ballif BC, Shaffer LG, Graves TA, Wilson RK, Schwartz DC, Eichler EE (2010). A large and complex structural polymorphism at 16p12.1 underlies microdeletion disease risk. Nat Genet.

[38] Girirajan S, Rosenfeld JA, Cooper GM, Antonacci F, Siswara P, Itsara A, Vives L, Walsh T, McCarthy SE, Baker C, Mefford HC, Kidd JM, Browning SR, Browning BL, Dickel DE, Levy DL, Ballif BC, Platky K, Farber DM, Gowans GC, Wetherbee JJ, Asamoah A, Weaver DD, Mark PR, Dickerson J, Garg BP, Ellingwood SA, Smith R, Banks VC, Smith W (2010). A recurrent 16p12.1 microdeletion supports a two-hit model for severe developmental delay. Nat Genet.

[39] El-Maarri O, Abdul-Sater Z, Yehya A, Beresian J, Salem E, Kamar A, Baydoun S, Shibbani K, Soubra A, Bitar F, Nemer G (2012). Two Heterozygous Mutations in NFATC1 in a Patient with Tricuspid Atresia. PLoS One.

[40] Zhao W, Niu G, Shen B, Zheng Y, Gong F, Wang X, Lee J, Mulvihill JJ, Chen X, Li S (2013). High-resolution analysis of copy number variants in adults with simple-to-moderate congenital heart disease. Am J Med Genet A.

