# Supplementary material for: Chromosome microarray testing for patients with congenital heart defects reveals novel disease causing loci and high diagnostic yield
Source: BMC Genomics. 2014 Dec 17;15(1):1127. doi: 10.1186/1471-2164-15-1127 (PMC4378009; doi:10.1186/1471-2164-15-1127)
Supplement: Supplementary file 1 — Additional file 1: Supplementary data. (DOCX 4 MB) [file 12864_2014_6876_MOESM1_ESM.docx]

**Additional file**

**Chromosome Microarray Testing for Patients with Congenital Heart Defects Reveals Novel Disease Causing Loci and High Diagnostic Yield**

Juan Geng, Jonathan Picker, Zhaojing Zheng, Xiaoqing Zhang, Jian Wang, Fuki Hisama, David W. Brown, Mary P. Mullen, David Harris, Joan Stoler, Ann Seman, David T. Miller, Qihua Fu, Amy E. Roberts, Yiping Shen

**Methods**

**CNV evaluation**

Detected CNVs meeting the following criteria were selected for further analysis: 1) deletions ≥10kb; duplications ≥50kb; 2) not found in the control populations that have been cataloged in the Database of Genomic Variants (DGV); 3) less than 50% overlap with known segmental duplications (SD).

Following the ACMG standards and guideline for interpretation of copy number variants, the remaining CNVs were classified into four categories: pathogenic (P), pathogenic/recessive gene deletion (P, RGD), likely pathogenic (LP), and variants of uncertain significance (VOUS). For this study, only genes that function in a dominant manner that are within the pathogenic CNVs and likely pathogenic CNVs were investigated.

**Gene prioritization for novel CHD candidate gene identification**

We developed an analytic process by integrating various tools and data sources to prioritize the genes involved in detected CNVs (Figure S2). For the purpose of identifying novel disease causing genes, we excluded the CNVs with known key causative genes (such as *TBX1* for 22q11.2) for further gene prioritization analysis. RefSeq genes encompassed in the pathogenic CNVs and likely pathogenic CNVs were assembled as the starting gene list.

First we used the Endeavour (http://www.esat.kuleuven.be/endeavour) and the ToppGene suite (<http://toppgene.cchmc.org/>) to independently rank all genes based on functional similarity to a training gene set consisting of 60 genes known to be involved in heart morphogenesis (Table S10). A cutoff threshold of p<0.05 was used for both analyses. Genes shared by two prioritization tools were retained for further assessment. Next we examined the gene expression pattern during mouse heart development using a combination of three databases: Gene Expression Database (GXD) in Mouse Genome Informatics (MGI) (http://www.informatics.jax.org/expression.shtml), Eurexpress (<http://www.eurexpress.org/ee/>) and Genepaint (<http://www.genepaint.org/Frameset.html>) for *in situ* expression in mice. CHD candidate genes were further narrowed based on their positive expression in endothelium, heart, or valves during heart development.

We next evaluated the resulting gene list with IPA tools to assess the enrichment of genes in cardiovascular system development.

**Results**

**Identification of novel CHD candidate genes**

Among 57 CNV regions of interest, ten CNVs contained genes known to be causal for CHD (Figure S1; Table S4). In order to identify novel CHD candidate genes, we examined the genes within the remaining 47 loci (Table S5). Starting from 647 genes in deletion CNVs and 517 genes in duplication CNVs, we performed a gene prioritization process using Endeavour and ToppGene. We next evaluated the expression patterns of shared genes by these two analyses in mouse embryonic heart. As a result, 123 and 289 genes in deletion CNVs reached statistical significance in Endeavour and ToppGene respectively. A large fraction of those genes (93 genes) were shared by the two analyses. Similarly, 96 and 140 genes in duplication CNVs were identified as significantly associated with heart morphogenesis by Endeavor and ToppGene respectively. Fifty-three of these genes were shared by the two analyses.

We next evaluated the expression patterns of these genes in mouse embryonic heart. As a result, 37 genes in deletion CNVs and 24 genes in duplication CNVs (Table S11) were found to be expressed in the mouse heart during development. The resulting gene lists were then subjected to Ingenuity Pathway Analysis (IPA). IPA demonstrated a significant enrichment of cardiovascular genes after the prioritization process. Specifically, for genes in deletion CNVs, the "Cardiovascular System Development and Function" pathway reached a p value of 1.58×10^-9^ after prioritization, which was more significant than before prioritization (2.19x10^-3^). Similarly, for genes in duplication CNVs, the p value before and after prioritization were 5.0x10^-4^ and 2.01x10^-8^ respectively for the "Cardiovascular System Development and Function" pathway. These data demonstrated the effectiveness of the enrichment and prioritization process.

The IPA analysis further narrowed the candidate gene targets and lead to the identification of 18 genes in deletion CNVs and 18 genes in duplication CNVs in the category of "Cardiovascular System Development and Function" (Figure S2, Table S6).

Furthermore, the same gene prioritization process was performed for individual cases carrying pathogenic CNVs of unknown CHD significance. No candidate gene was identified in four cases with CNVs smaller than 300kb, and a total of 39 genes were identified in the remaining 19 cases (Table S7). Interestingly 20 of these genes were also contained in the global prioritization list (bold genes in Table S7). These shared genes are considered to be the most likely dosage sensitive novel CHD candidate genes.

**Table S1**. Fifty-eight pathogenic CNVs

| **Locus** | **Coordinates (hg 19)** | **Size range (kb)** | **CN** | | **No of cases** | **Key causal gene(s)** |
| --- | --- | --- | --- | --- | --- | --- |
| **BCH (50 CNVs)** | | | | | | |
| 22q11.2 | 18890271-21464060 | 2,500 | Loss | 4 | | *TBX1* |
|  | 18890271-21505358 | 2,600 |  |  |  |  |
|  | 18900473-21461788 | 2,600 |  |  |  |  |
|  | 18706001-21505358 | 2,750 |  |  |  |  |
|  | 18706001-21505358 | 2,800 | Gain | 3 | |  |
|  | 18900473-21797516 | 2,900 |  |  |  |  |
|  | 18706001-21801661 | 3,000 |  |  |  |  |
| 8p23.1 | 7370178-11803911 | 4,500 | Loss | 3 | | *GATA4* |
|  | 7053186-12241152 | 5,200 |  |  |  |  |
|  | 183052-25069723 | 24,900 |  |  |  |  |
|  | 8079861-11866551 | 3,800 | Gain | 2 | |  |
|  | 3981625-15431384 | 11,500 |  |  |  |  |
| 4q terminal | 185910060-190469278 | 4,600 | Loss | 4 | | N |
|  | 178260978-190790881 | 12,500 |  |  |  |  |
|  | 178260978-190790822 | 12,500 |  |  |  |  |
|  | 171166053-190435113 | 19,300 |  |  |  |  |
| 7q35-q36.3 | 156393046-158821316 | 2,400 | Loss | 2 | | N |
|  | 146705271-159118507 | 12,000 |  |  |  |  |
| 14q32.31-q32.33 | 102199089-107189890 | 4,900 | Gain | 2 | | N |
|  | 102199089-107278711 | 5,100 |  |  |  |  |
| 15q13.2-q13.3 | 30943903-32462642 | 1,500 | Gain | 2 | | N |
|  | 30943903-32861567 | 1,900 |  |  |  |  |
| 1q21.1-q21.2 | 145736237-149140953 | 3,400 | Gain | 1 | | N |
| 1q41-q42.12 | 223521035-226332649 | 2,800 | Loss | 1 | | *LEFTY2* |
| 1q43-q44 | 241864236-249212879 | 7,300 | Loss | 1 | | N |
| 2p25.3-p23.2 | 202591-29213023 | 29,000 | Gain | 1 | | N |
| 3p21.31 | 46023015-48112638 | 2,080 | Loss | 1 | | *TDGF1* |
| 3p26.3-p25.2 | 64066-12093306 | 12,000 | Loss | 1 | | *CRELD1* |
| 4p16.3-p15.31 | 56772-23210956 | 23,000 | Gain | 1 | | *EVC, EVC2* |
| 5q14.3 | 89735448-89992221 | 257 | Loss | 1 | | N |
| 6q24.3-q25.1 | 148861122-152317713 | 3,400 | Loss | 1 | | *TAB2* |
| 6q25.3-q27 | 157531075-170895991 | 13,400 | Gain | 1 | | N |
| 8p21.3-p21.1 | 20711878-27383826 | 6,700 | Loss | 1 | | *NKX2-6* |
| 8p23.3-p23.2 | 176464-4103422 | 3,900 | Gain | 1 | | N |
| 8q21.11-q22.1 | 75130234-96660340 | 21,437 | Loss | 1 | | N |
| 9p24.3-p23 | 204193-9943399 | 9,700 | Gain | 1 | | N |
| 9q21.13-q21.31 | 77882055-82079160 | 4,200 | Loss | 1 | | N |
| 9q22.2-q22.33 | 93184413-99954824 | 6,800 | Loss | 1 | | N |
| 9q34.11-q34.3 | 132197825-141025921 | 8,900 | Gain | 1 | | N |
| 9q34.3 | 139259791-141073897 | 1,800 | Loss | 1 | | *NOTCH1* |
| 10q26.13-q26.3 | 124461578-135474787 | 10,800 | Loss | 1 | | N |
| 11q24.2-q25 | 124763109-134927055 | 10,200 | Loss | 1 | | N |
| 13q33.2-q34 | 105856761-115169878 | 9,300 | Loss | 1 | | N |
| 15q11.2-q13.1 | 22669052-29030517 | 6,600 | Loss | 1 | | N |
| 16p11.2 | 28824794-29044717 | 220 | Loss | 1 | | N |
| 17p13.3 | 1-1692338 | 1,600 | Loss | 1 | | N |
| 17q12 | 34815184-36248918 | 1,400 | Gain | 1 | | N |
| 18p11.32-p11.31 | 14316-3776254 | 3,800 | Loss | 1 | | N |
| 19p13.12-p13.2 | 7506482-14218238 | 6,600 | Gain | 1 | | N |
| 22q11.1-q11.21 | 17059296-18641479 | 1,600 | Gain | 1 | | N |
| **SCMC (8 CNVs)** | | | | | | |
| 22q11.21 | 18916842-21041014 | 2,124 | Loss | 5 | | *TBX1* |
|  | 18916842-21465662 | 2,400 |  |  |  |  |
|  | 18916842-21800797 | 2,800 |  |  |  |  |
|  | 18916842-21798907 | 2,882 |  |  |  |  |
|  | 18916842-21798907 | 2,882 |  |  |  |  |
| 4q terminal | 182274314-190957473 | 8,600 | Loss | 1 | | N |
| 5p15.33-p15.32 | 113576-4726683 | 4,600 | Loss | 1 | | *IRX4* |
| 18q22.3-q23 | 68400575-78014123 | 9,611 | Loss | 1 | | N |

N: Unknown

**Table S2.** Thirty-one likely pathogenic CNVs

| **Locus** | **Coordinates (hg 19)** | **Size range (kb)** | **CN** | **No of cases** |
| --- | --- | --- | --- | --- |
| **BCH (28 likely pathogenic CNVs)** | | | | |
| 15q11.2 | 22842165-23087552 | 245 | Loss | 3 |
|  | 22669052-23221690 | 553 |  |  |
|  | 21265266-23228393 | 2,000 |  |  |
| 16p12.1 | 21951379-22430592 | 479 | Loss | 3 |
|  | 21951223-22430623 | 479 |  |  |
|  | 21951379-22430533 | 480 |  |  |
| Yp11.2 | 5696701-6969048 | 1,300 | Loss | 3 |
|  | 6397810-9131638 | 2,733 |  |  |
|  | 6414449-9168616 | 3,000 |  |  |
| 16p12.2-p12.1 | 21599687-21837492 | 238 | Loss | 2 |
|  | 21599687-21837551 |  |  |  |
| 2q31.2 | 178889682-179516322 | 627 | Gain | 1 |
| 5q12.1-q12.2 | 60645736-63657348 | 3,000 | Gain | 1 |
| 6q26 | 162627660-162757221 | 130 | Loss | 1 |
| 8p23.1 | 6360178-6429522 | 69 | Loss | 1 |
| 8p23.2 | 2759193-4146595 | 1,400 | Gain | 1 |
| 9p21.1 | 28562085-32251430 | 3,700 | Gain | 1 |
| 10q21.3 | 66134296-67914417 | 1,800 | Gain | 1 |
| 10q24.2 | 100723330-100909863 | 187 | Loss | 1 |
| 11p11.2 | 44180352-44193814 | 13 | Loss | 1 |
| 11q14.1-q14.2 | 85272655-86503862 | 1,200 | Loss | 1 |
| 13q12.12 | 23553332-24910765 | 1,358 | Loss | 1 |
| 15q26.1 | 89804975-89841112 | 37 | Loss | 1 |
| 16p13.3 | 7026156-7281364 | 255 | Loss | 1 |
| 18q21.32 | 57997060-58067629 | 71 | Gain | 1 |
| 19q13.33 | 51857827-51926276 | 69 | Loss | 1 |
| 22q11.21-q11.22 | 21806675-22444158 | 637 | Loss | 1 |
| 22q12.1 | 28887136-29130362 | 244 | Loss | 1 |
| **SCMC (3 CNVs)** | | | | |
| 15q11.2 | 22770421-23282905 | 512 | Loss | 1 |
| 6q26 | 162625549-162799405 | 174 | Loss | 1 |
| 13q12.12 | 23504907-24910415 | 1,406 | Loss | 1 |

**Table S3.** Classification of CHD phenotypes.

|  | **CHD categories** | **Primary Phenotypes** |
| --- | --- | --- |
| A | Septal defects and mild PS | ASD, VSD, mild PS |
| B | Isolated abnormities of valves | BAV, TR, MR, PR, AR |
| C | Obstruction of right ventricular outflow tract caused by abnormities of valves | TS, TA, Severe PS with hypoplastic right ventricular, PA/IVS |
| D | Obstruction of left ventricular outflow tract | MS, AS, COA, IAA |
| E | Isolated conotruncal defects | TOF, DORV, TGA, PTA, PA/VSD |
| F | Compound conotruncal defects | TOF/DORV/TGA/PTA/PA with other heart defects |
| G | HLHS |  |
| H | Heterotaxy syndrome |  |
| I | Others | CAVC, TAPVC, SV, etc. |

This categorization was performed according to a combination of the standards defined in “Nomenclature and classification of congenital cardiac surgery” and the classification system established by NBDPS.

The following abbreviations were used: ASD, atrial septal defect; AR, aortic regurgitation; AS, aortic stenosis; BAV, bicuspid aortic valve; CAVC, complete atrioventricular canal; CoA, coarctation of the aorta; DORV, double outlet right ventricle; HLHS, hypoplastic left heart syndrome; IAA, interruption of aortic arch; MR, mitral regurgitation; MS, mitral stenosis; PA, pulmonary atresia; PA/IVS, pulmonary atresia with intact ventricular septum; PS, pulmonic stenosis; PTA, persistent truncus arteriosus; SV, single ventricle heart defect; TA, tricuspid atresia; TAPVC, total anomalous pulmonary venous connection; TGA, transposition of the great arteries; TOF, tetralogy of fallot; TR, tricuspid regurgitation; TS, tricuspid stenosis; VSD, ventricular septal defect.

**Table S4**. Ten chromosomal loci containing gene(s) known to be associated with CHD

|  | **Chromosomal locus** | **Reported CHD-causing genes** |
| --- | --- | --- |
| 1 | 1q41-q42.12 | *LEFTY2* |
| 2 | 3p21.31 | *TDGF1* |
| 3 | 3p26.3-p25.2 | *CRELD1* |
| 4 | 4p16.3-p15.31 | *EVC, EVC2* |
| 5 | 5p15.33-p15.32 | *IRX4* |
| 6 | 6q24.3-q25.1 | *TAB2* |
| 7 | 8p21.3-p21.1 | *NKX2-6* |
| 8 | 8p23.1 | *GATA4* |
| 9 | 9q34.3 | *NOTCH1* |
| 10 | 22q11.21 | *TBX1* |

**Table S5.** Forty-seven candidate genomic loci

|  | **Chromosome** | **CN** | **Size(kb)** | **Coordinates (hg19)** |
| --- | --- | --- | --- | --- |
| 1 | 1q21.1-q21.2 | Gain | 3,400 | chr1:145736237-149140953 |
| 2 | 1q43-q44 | Loss | 7,300 | chr1:241864236-249212879 |
| 3 | 2p25.3-p23.2 | Gain | 29,000 | chr2:202591-29213023 |
| 4 | 2q31.2 | Gain | 627 | chr2:178889682-179516322 |
| 5 | 4q35.1-q35.2 | Loss | 4,600 | chr4:185910060-190469278 |
| 6 | 5q12.1-q12.2 | Gain | 3,000 | chr5:60645736-63657348 |
| 7 | 5q14.3 | Loss | 257 | chr5:89735448-89992221 |
| 8 | 6q25.3-q27 | Gain | 13,400 | chr6:157531075-170895991 |
| 9 | 6q26 | Loss | 174 | chr6:162625549-162799405 |
| 10 | 7q35-q36.3 | Loss | 12,000 | chr7:146705271-159118507 |
| 11 | 8p23.1 | Loss | 69 | chr8:6360178-6429522 |
| 12 | 8p23.2 | Gain | 1,400 | chr8:2759193-4146595 |
| 13 | 8p23.3-p23.2 | Gain | 3,900 | chr8:176464-4103422 |
| 14 | 8q21.11-q22.1 | Loss | 21,437 | chr8:75130234-96660340 |
| 15 | 9p21.1 | Gain | 3,700 | chr9:28562085-32251430 |
| 16 | 9p24.3-p23 | Gain | 9,700 | chr9:204193-9943399 |
| 17 | 9q21.13-q21.31 | Loss | 4,200 | chr9:77882055-82079160 |
| 18 | 9q22.2-q22.33 | Loss | 6,800 | chr9:93184413-99954824 |
| 19 | 10q21.3 | Gain | 1,800 | chr10:66134296-67914417 |
| 20 | 10q24.2 | Loss | 187 | chr10:100733340-100919873 |
| 21 | 10q26.13-q26.3 | Loss | 10,800 | chr10:124461578-135474787 |
| 22 | 11p11.2 | Loss | 13 | chr11:44180352-44193814 |
| 23 | 11q14.1-q14.2 | Loss | 1,200 | chr11:85272655-86503862 |
| 24 | 11q24.2-q25 | Loss | 10,200 | chr11:124763109-134927055 |
| 25 | 13q12.12 | Loss | 1,406 | chr13:23504907-24910415 |
| 26 | 13q33.2-q34 | Loss | 9,300 | chr13:105856761-115169878 |
| 27 | 14q32.31-q32.33 | Gain | 5,100 | chr14:102199089-107278711 |
| 28 | 15q11.2 | Loss | 2,000 | chr15:21265266-23228393 |
| 29 | 15q11.2-q13.1 | Loss | 6,600 | chr15:22669052-29030517 |
| 30 | 15q13.2-q13.3 | Gain | 1,900 | chr15:30943903-32861567 |
| 31 | 15q26.1 | Loss | 37 | chr15:89804975-89841112 |
| 32 | 16p11.2 | Loss | 220 | chr16:28824794-29044717 |
| 33 | 16p12.1 | Loss | 480 | chr16:21951379-22430533 |
| 34 | 16p12.2-p12.1 | Loss | 238 | chr16:21599687-21837551 |
| 35 | 16p13.3 | Loss | 255 | chr16:7026156-7281364 |
| 36 | 17p13.3 | Loss | 1,600 | chr17:1-1692338 |
| 37 | 17q12 | Gain | 1,400 | chr17:34815184-36248918 |
| 38 | 18p11.32-p11.31 | Loss | 3,800 | chr18:14316-3776254 |
| 39 | 18q21.32 | Gain | 71 | chr18:57997060-58067629 |
| 40 | 18q22.3-q23 | Loss | 9,611 | chr18:68400575-78014123 |
| 41 | 19p13.12-p13.2 | Gain | 6,600 | chr19:7506482-14218238 |
| 42 | 19q13.33 | Loss | 69 | chr19:51857827-51926276 |
| 43 | 22q11.1-q11.21 | Gain | 1,600 | chr22:17059296-18641479 |
| 44 | 22q11.21-q11.22 | Loss | 637 | chr22:21806675-22444158 |
| 45 | 22q12.1 | Loss | 244 | chr22:28887136-29130362 |
| 46 | Yp11.2 | Loss | 1,300 | chrY:5696701-6969048 |
| 47 | Yp11.2 | Loss | 3,000 | chrY:6414449-9168616 |

**Table S6.** Summary of novel candidate genes after prioritization procedures.

|  | **Candidate Genes** |
| --- | --- |
| Deletions | *ANGPT2, COL4A1, CRK, CTBP2, EFNB2, ETS1, F7, FAT1, FLI1, HEY1, KCNH2, MAPK1, NFATC1, NOS3, PTCH1, SERPINF1, SHH, SORBS2* |
| Duplications | *CALR, CNN1, DLL1, ELAVL1, EPOR,* ***GJA5****, HNRNPM, ID2, IGF2R, JAK2, MYCN, PRKACA, QKI, RHOB, RPS6KA2, SMARCA4, TTN, YWHAQ* |

By evaluating the expression patterns of loss genes and gain genes confirmed through Endeavour & ToppGene in mouse embryonic heart and performing IPA core analysis, numerous potential candidate genes involved in cardiovascular development were identified.

The gene in bold was previously identified to be associated with CHD.

**Table S7.** Summary of candidate CHD genes in individual patient with non-recurrent pathogenic CNV(s)

| **Case No.** | **Locus (hg19)** | **CNV** | **Size(kb)** | **Inheritance** | **Cardiac Phenotypes** | **Candidate genes** |
| --- | --- | --- | --- | --- | --- | --- |
| 2 | 16p11.2 | Loss | 220 | N/a | D-TGA, AV canal type VSD, MA | N |
| 4 | 4q35.1-q35.2 | Loss | 4600 | N/a | DILV, hypoplastic aortic arch, CoA | *SLC25A4* |
| 5 | 18p11.32-p11.31 | Loss | 3800 | N/a | Double inlet, double outlet single right ventricle, Hypoplastic mitral valve, hypoplastic left ventricle, IAA | *TGIF1, THOC1* |
| 31 | 2p25.3-p23.2 | Gain | 29000 | N/a | Subaortic stenosis | ***MYCN*** |
| 43 | 17p13.3 | Loss | 1600 | de novo | TOF | ***CRK,*** *YWHAE* |
| 46 | 17q12 | Gain | 1400 | familial | TOF, subvalvar pulmonary stenosis | *HNF1B* |
| 51 | 9q21.13-q21.31 | Loss | 4200 | N/a | ASD, peripheral PS | *PCSK5* |
| 56 | 9q34.2 | Gain | 146 | de novo | TA, IAA, VSD, | N |
| 66 | 19p13.12-p13.2 | Gain | 6600 | de novo | ASD, PDA, PS | ***EPOR, CALR, CNN1*** |
| 67 | 18q22.3-q23 | Loss | 9611 | N/a | TOF/ASD | ***NFATC1*** |
| 70 | 17p13.2 | Loss | 51 | familial | DORV with right ventricle hypoplasia, VSD, PS | *NOTCH1, ABL1, RXRA* |
|  | 8p23.1 | loss | 69 | familial |  |  |
|  | 9q34.11-q34.3 | Gain | 8900 | familial |  |  |
|  | 10q26.13-q26.3 | Loss | 10800 | de novo |  |  |
| 71 | 9p24.3-p23 | Gain | 9700 | N/a | TA,VSD | ***EFNB2, JAK2, F7*** |
|  | 13q33.2-q34 | Loss | 9300 | N/a |  |  |
| 74 | 15q11.2-q13.1 | Loss | 6600 | N/a | ASD, AV canal | *NDN* |
| 83 | 16q24.3 | Loss | 137 | de novo | transitional AV canal, ASD, cleft mitral valve | N |
| 85 | 15q13.2-q13.3 | Gain | 1900 | familial | ASD, PAPVC | *KLF13* |
| 90 | 4p16.3-p15.31 | Gain | 23000 | N/a | TOF/PA | *CTBP1, FGFR3,* ***KCNH2****, MSX1,* ***NOS3, SHH,*** *SLIT2* |
|  | 7q35-q36.3 | Loss | 12000 | N/a |  |  |
| 94 | 11q24.2-q25 | Loss | 10200 | N/a | VSD, CoA, AS, small left-sided structures, a large PDA, leftward malrotation of the atrial septal | ***FLI1, ETS1*** |
| 100 | 9q22.2-q22.33 | Loss | 6800 | de novo | ASD, VSD | *ROR2,* ***PTCH1*** |
| 106 | 8q21.11-q22.1 | Loss | 21437 | N/a | multiple VSDs | ***HEY1*** |
| 129 | 1q43-q44 | Loss | 7300 | N/a | VSD, sub AS, DORV, mild LV hypoplasia, MS | ***DLL1, QKI, RPS6KA2****, AKT3, THBS2* |
|  | 6q25.3-q27 | Gain | 13400 | N/a |  |  |
| 130 | 22q11.1-q11.21 | Gain | 1600 | N/a | TAPVR, ASD | *BID* |
| 131 | 1q21.1-q21.2 (hg18:1q21.1) | Gain | 3400 | N/a | TOF/PA, MAPCAs | ***GJA5*** |
| 133 | 5q14.3 | Loss | 257 | N/a | PA, nearly intact ventricular septum with a small VSD | N |

N: not identified. Genes in in bold were also identified in globe prioritization process. Genes underlined were previously identified as causative for CHD.

**Table S8.** Summary of the contribution of rare genic CNVs to the CHD pathogenesis from literature

|  | **Study** | **Platform** | **Patients** | | **Non-polymorphic CNVs (%)** |
| --- | --- | --- | --- | --- | --- |
|  |  |  | **No.** | **phenotype** |  |
| 1 | Thienpont B, et al (2007) [[1](#_ENREF_1)] | 1 Mb BAC/PAC | 60 | syndromic CHD | 18 (30%) |
| 2 | Greenway SC, et al (2009) [[2](#_ENREF_2)] | Affymetrix 6.0 array | 114 | isolated TOF | 11(10%) |
| 3 | Breckpot J, et al (2010) [[3](#_ENREF_3)] | 1 Mb BAC/PAC | 90 | syndromic CHD | 28 (31.1%) |
| 4 | Lalani SR, et al (2012) [[4](#_ENREF_4)] | Agilent customized 105K CGH array | 203 | syndromic CHD | 70 (34.5%) |
| 5 | Soemedi R, et al (2012) [[5](#_ENREF_5)] | Illumina 660W-Quad SNP array | 2256 | isolated CHD | 16.50% |
| 6 | Warburton D, et al (2014) [[6](#_ENREF_6)] | NimbleGen HD2-2.1 CGH | 93 | isolated CTD or HLHS | 22 (23.7%) |
|  |  |  | 108 | CTD or HLHS with extracardiac anomaly | 23 (21.3%) |

*CTD: conotruncal defect

**Table S9**. Summary of 20 novel CHD candidate genes

|  | | **Expression** | | **Phenotypic Alleles in MGI** | |  |
| --- | --- | --- | --- | --- | --- | --- |
|  |  | **RNA In situ** | **Others*** | **Category** | **Abnormal phenotypes reported in cardiovascular System** |  |
| **Deletions** | | | | | |  |
| 1 | *Crk* |  | Present (P adult) | Targeted (knock-out) | abnormal heart ventricle morphology (dilated heart left ventricle, dilated heart right ventricle, thin ventricular wall) |  |
| 2 | *Efnb2* | Weak (E 14.5) |  | Targeted (knock-out) | abnormal cardiovascular development, abnormal heart morphology |  |
| 3 | *Ets1* | Strong (E14.5) |  | Targeted (knock-out) | abnormal vascular branching morphogenesis, dilated vasculature, abnormal vascular endothelial cell physiology |  |
| 4 | *F7* |  | Moderate (P W 6-8) | Targeted (knock-out) | abnormal dorsal aorta morphology |  |
| 5 | *Fli1* | Moderate (E14.5) |  | Targeted (knock-out) | abnormal vascular development |  |
| 6 | *Hey1* | Present (E 8.5, 9.5, 10.5) |  | Targeted (knock-out) | abnormal atrioventricular cushion morphology, abnormal interventricular septum morphology |  |
| 7 | *Kcnh2* | Present (E 13.5) |  | Targeted (knock-out) | decreased heart rate |  |
| 8 | *Nfatc1* | Strong (E 9.5, E 10.5) |  | Targeted (knock-out) | abnormal heart morphology (VSD, abnormal semilunar valve morphology), abnormal outflow tract development |  |
| 9 | *Nos3* | Moderate (E 14.5) |  | Targeted (knock-out) | decreased heart rate, hypertension, increased vasoconstriction |  |
| 10 | *Ptch1* | Present (E 10.5) |  | Targeted (knock-out) | N |  |
| 11 | *Shh* | Weak (E 14.5) | Present (E 10, 10.5) | Targeted (knock-out) | abnormal outflow tract development (PTA), abnormal artery morphology, failure of heart looping |  |
| **Duplications** | | | | | | |
| 1 | *Calr* |  | Present (E 18.0) | Targeted (knock-in) | N |  |
| 2 | *Cnn1* | Strong (E 9.5, 13.5) |  | Targeted (knock-in) | N |  |
| 3 | *Dll1* | Moderate to Strong (E 14.5) |  | Targeted (knock-in) | abnormal blood vessel morphology (AS) |  |
| 4 | *Epor* | Moderate (E 10.5) Present (E 12.5) |  | Targeted (knock-in) | N |  |
| 5 | *Gja5* | Moderate (E 14.5) |  | Targeted (knock-in) | abnormal impulse conducting system conduction |  |
| 6 | *Jak2* |  | Moderate (P W 6-8) | Targeted (knock-in) | N |  |
| 7 | *Mycn* | Present (E 9.5, 10.5, 12.5) |  | Targeted (knock-in) | abnormal angiogenesis, absent vitelline blood vessels, thin myocardium |  |
| 8 | *Qki* | moderate (E14.5) |  | Chemically induced (ENU) single point mutation | abnormal heart looping |  |
| 9 | *Rps6ka2* | Strong (E14.5) |  | N | N |  |

* Other expression data is from the following assay types: immunohistochemistry, RT-PCR, Northern and Western blots, etc.

P adult: postnatal adult

Phenotypes with underline are associated with CHD.

**Table S10.** Sixty genes in training set

| Genes | Chromosome location | Phenotypes | OMIM |
| --- | --- | --- | --- |
| *ACTC1* | 15q14 | ASD | 102540 |
| *ACVR1* | 2q23-q24 | AVSD | 102576 |
| *ACVR2B* | 3p22 | PS, DORV, TGA, Heterotaxy | 602730 |
| *ALDH1A2* | 15q21.3 | TOF | 603687 |
| *ANKRD1* | 10q23.31 | TAPVR | 609599 |
| *BRAF* | 7q34 | ASD, PS, TOF, Noonan syndrome | 164757 |
| *CBL* | 11q23.3 | PS, ASD, VSD, Noonan syndrome | 165360 |
| *CFC1* | 2q21.1 | TOF, TGA, AVSD, ASD, VSD, IAA, DORV | 605194 |
| *CHD7* | 8q12.2 | PDA, TOF, AVSD, HLHS, DORV, ASD, VSD, RV hypoplasia, CHARGE syndrome | 608892 |
| *CITED2* | 6q23.3 | ASD, VSD | 602937 |
| *CRELD1* | 3p25.3 | ASD, AVSD | 607170 |
| *CTNND2* | 5p15.2 | VSD, PDA, ASD, TOF, Cri-Du-Chat syndrome | 604275 |
| *DVL1* | 1p36 | PDA | 601365 |
| *ELN* | 7q11.23 | PS, AS, BAV, Williams-Beuren syndrome | 130160 |
| *EVC* | 4p16 | ASD, Ellis-van Creveld syndrome | 604831 |
| *EVC2* | 4p16.2-p16.1 | VSD, PDA, AVSD, ASD, Ellis-van Creveld syndrome | 607261 |
| *FBN1* | 15q21.1 | MR, Mitral valvar prolapse, Marfan syndrome | 134797 |
| *FLNA* | Xq28 | AS, MA, AR, PDA, CoA | 300017 |
| *FOXH1* | 8q24.3 | TOF, TGA | 603621 |
| *GATA4* | 8p23.1-p22 | ASD, PS, VSD, TOF, AVSD, PAPVR | 600576 |
| *GATA6* | 18q11.1-q11.2 | ASD, TOF, PS, AVSD, PDA, VSD | 601656 |
| *GDF1* | 19p12 | Heterotaxy, TOF, TGA, DORV | 602880 |
| *GJA1* | 6q22.31 | ASD, HLHS, TAPVR | 121014 |
| *HAND2* | 4q33 | TOF | 602407 |
| *HRAS* | 11p15.5 | ASD, VSD, PS, Costello syndrome | 190020 |
| *IRX4* | 5p15.3 | VSD | 606199 |
| *JAG1* | 20p12.1-p11.23 | TOF, VSD, PS, PA, AS, Algille syndrome | 601920 |
| *KMT2D (MLL2)* | 12q13.12 | VSD, ASD, TOF, SV, CoA, PDA, TGA, Kabuki syndrome | 602113 |
| *KRAS* | 12p12.1 | PS, ASD, VSD, Cardiofaciocutaneous syndrome | 190070 |
| *LEFTY2* | 1q42.1 | TGA, AVSD, IAA, CoA, L-R Axis defects | 601877 |
| *MAP2K1* | 15q22.1-q22.33 | PS, TOF, PAPVC, ASD, Cardiofaciocutaneous syndrome | 176872 |
| *MAP2K2* | 19p13.3 | PS, BAV, ASD, Cardiofaciocutaneous syndrome | 601263 |
| *MED13L(THRAP2)* | 12q24.21 | TGA | 608771 |
| *MYH11* | 16p13.11 | PDA, Aortic aneurysm | 160745 |
| *MYH6* | 14q12 | ASD, TA, AS, PFO, TGA | 160710 |
| *MYH7* | 14q12 | Ebstein anomaly, ASD | 160760 |
| *NF1* | 17q11.2 | CoA, PS, Noonar syndrome | 613113 |
| *NKX2-5* | 5q34 | ASD, VSD, TOF, HLHS, CoA, TGA, DORV, IAA | 600584 |
| *NKX2-6* | 8p21.2 | PTA | 611770 |
| *NODAL* | 10q22.1 | TGA, PA, TOF, DORV, dextrocardia, TAPVR, AVSD | 601265 |
| *NOTCH1* | 9q34.3 | BVA, AS, CoA, HLHS | 190198 |
| *NOTCH2* | 1p13-p11 | TOF, PS, Peripheral pulmonary hypoplasia, Alagille syndrome | 600275 |
| *NRAS* | 1p13.2 | PS, ASD, VSD, Noonar syndrome | 164790 |
| *PDGFRA* | 4q12 | TAPVR | 173490 |
| *PTPN11* | 12q24 | VSD, CoA, PS, AVSD, ASD, AS, Noonar syndrome | 176876 |
| *RAF1* | 3p25 | PS, Hypertrophic cardiomyopathy, Noonar syndrome | 164760 |
| *SALL4* | 20q13.2 | VSD, PFO, TOF, Duane-radial ray syndrome | 607343 |
| *SEMA3E* | 7q21.11 | TOF, ASD, VSD, CHARGE syndrome | 608166 |
| *SHOC2* | 10q25 | ASD, PS, VSD, Noonar syndrome | 602775 |
| *SMAD6* | 15q21-q22 | BAV, CoA, AS | 602931 |
| *SOS1* | 2p21 | ASD, VSD, AS, PS, Noonar syndrome | 182530 |
| *TAB2* | 6q25.1 | LV outflow tract obstruction | 605101 |
| *TBX1* | 22q11.21 | TOF, PS, IAA | 602054 |
| *TBX20* | 7p14.3 | ASD, MS, VSD | 606061 |
| *TBX5* | 12q24.1 | ASD,VSD, AVSD, DORV, TOF, PFO, Holt Oram syndrome | 601620 |
| *TDGF1* | 3p21.31 | TOF, VSD | 187395 |
| *TFAP2B* | 6p12 | PDA, BAV, VSD, CoA | 601601 |
| *VEGFA* | 6p12 | CoA | 192240 |
| *ZFPM2(FOG2)* | 8q23 | TOF, DORV, TA | 603693 |
| *ZIC3* | Xq26.2 | TGA, PS, DORV, TAPVR, ASD, HLHS, VSD, Dextrocadia, L-R axis defects | 300265 |

The above genes are compiled based on the review articles by Fahed et al.(2013) and Pierpont et al. (2007) [7, 8] and CHDWiki (http://homes.esat.kuleuven.be/~bioiuser/chdwiki/ index.php/Main_Page)

**Table S11.** Candidate genes with positive expression in mouse heart.

|  | **No. of genes** | **Identified Genes** |
| --- | --- | --- |
| **Deletions** | **37** | *ADNP2, ANGPT2, BARX2, CDK5, CHST15, COL4A1, CRK, CTBP2, DNAJB6, DPYSL4, E2F5, EFNB2, ETS1, EZH2, F7, FAT1, FLI1, HEY1, KCNH2, KCNJ1, MAPK1, MNX1, MYOM1, NCAPG2, NDN, NFATC1, NOS3, PHF2, PPM1F, PTCH1, RUNX1T1, SERPINF1, SHH, SORBS2, TFDP1, UBE2L3, YWHAE* |
| **Duplications** | **24** | *BID, CALR, CNN1, DLL1, E2F6, ELAVL1, EPOR, EZR, GJA5, HNF1B, HNRNPM, ID2, IGF2R, JAK2, MYCN, PRKACA, QKI, RHOB, RPS6KA2, SMARCA2, SMARCA4, TBP, TTN, YWHAQ* |


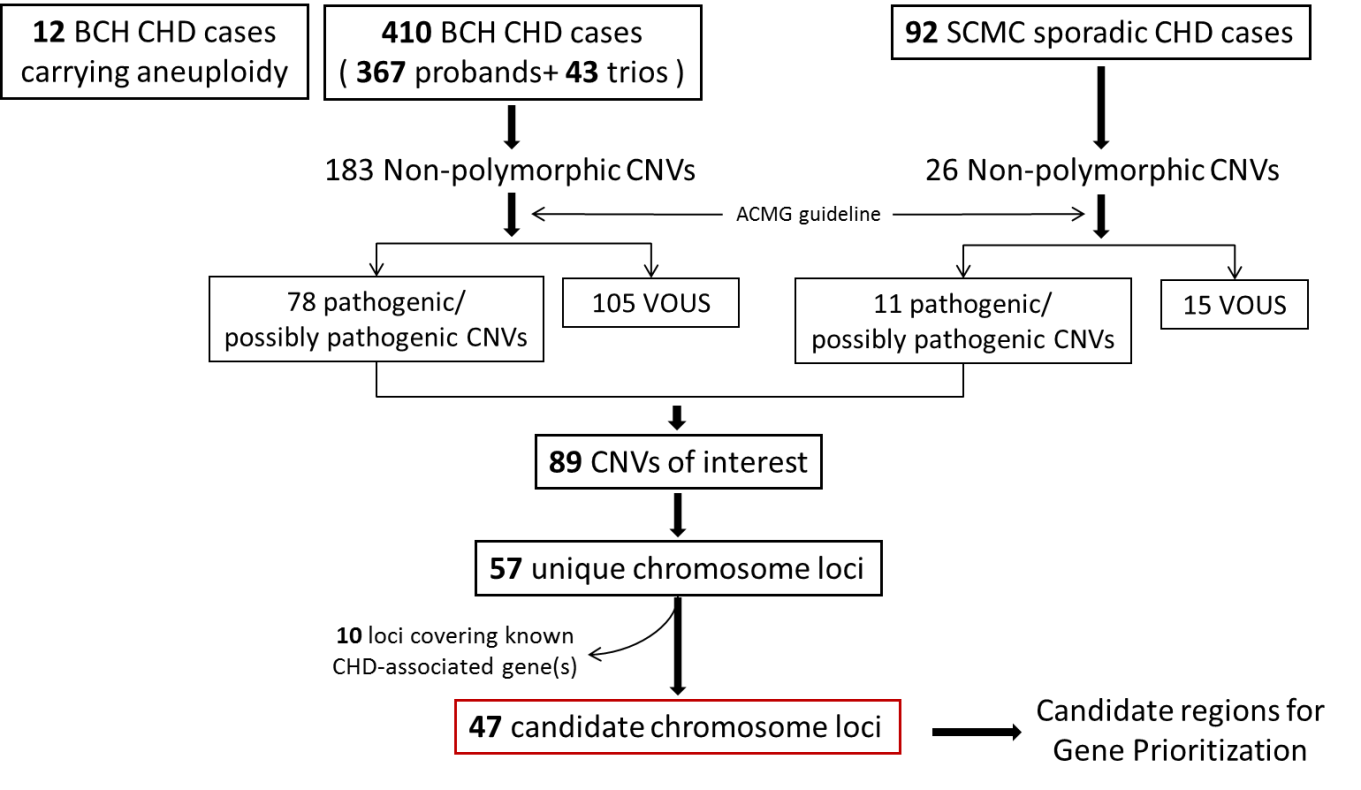


**Figure S1.** Analysis and filtering of CNVs.


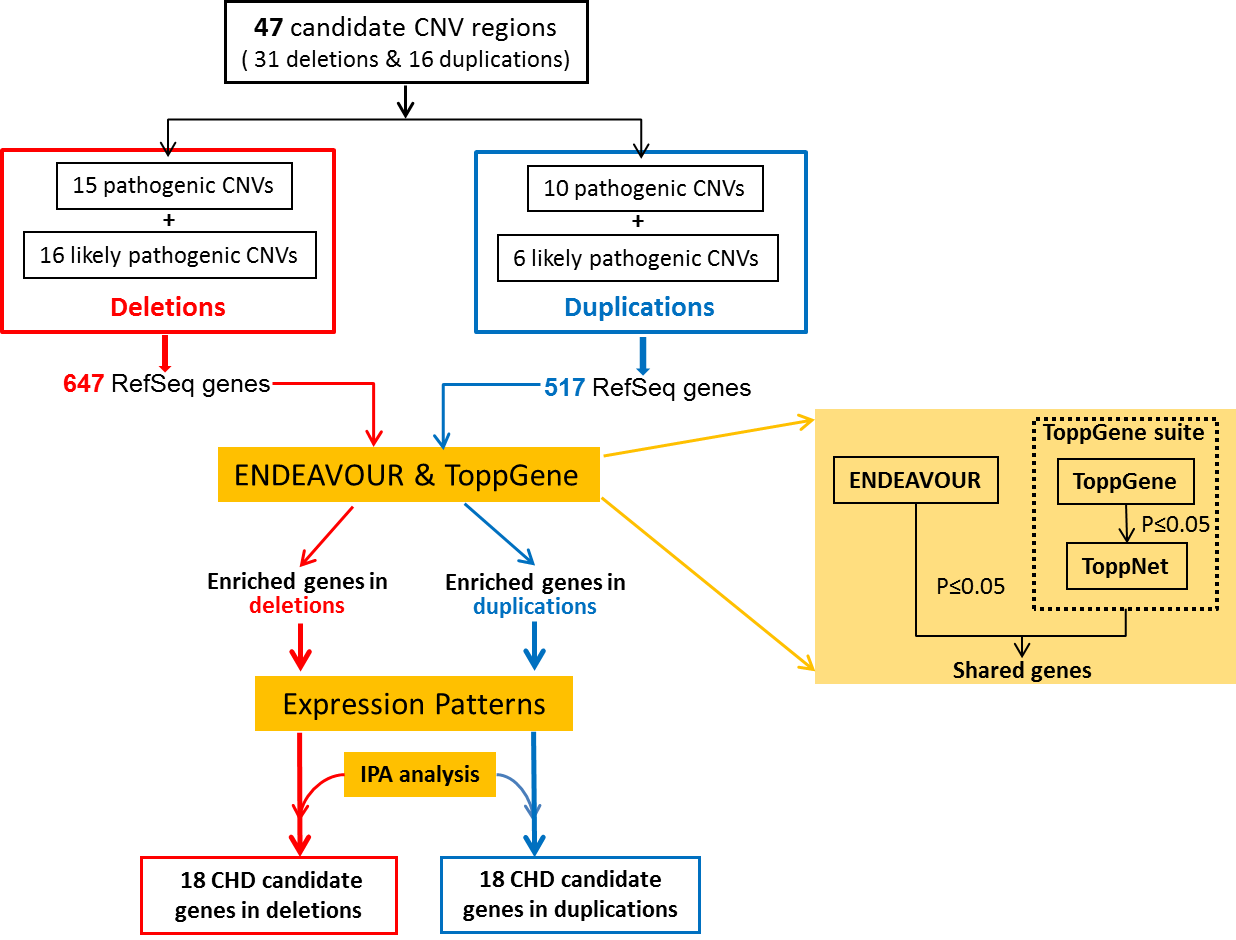


**Figure S2.** The gene prioritization process.


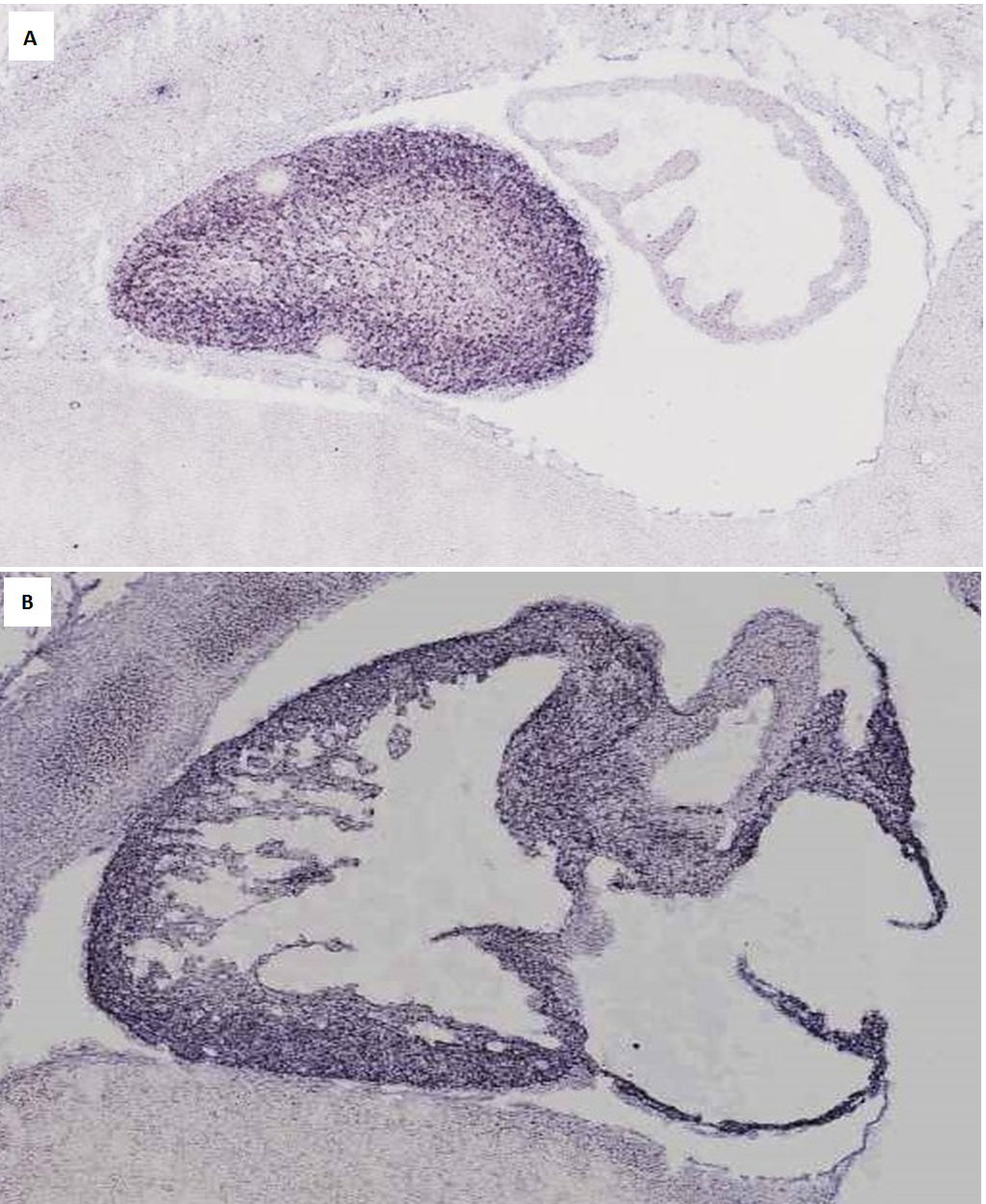


**Figure S3.** mRNA expression profile of *Sorbs2* and *Slc25a4* in mouse embryonic heart. (A) *In situ* hybridization for *Sorbs2* of a wild-type stage E 14.5 mouse heart, illustrating strong expression in epithelia and cardiac muscle tissue. (B) *In situ* hybridization for *Slc25a4* of a wild-type stage E 14.5 mouse heart. Pictures are taken from Eurexpress (<http://www.eurexpress.org/ee/>) and Genepaint (<http://www.genepaint.org/Frameset.html>).

**Reference**

1. Thienpont B, Mertens L, de Ravel T, Eyskens B, Boshoff D, Maas N, Fryns JP, Gewillig M, Vermeesch JR, K. D: **Submicroscopic chromosomal imbalances detected by array-CGH are a frequent cause of congenital heart defects in selected patients**. *Eur Heart J* 2007(22):2778-2784.

2. Greenway SC, Pereira AC, Lin JC, DePalma SR, Israel SJ, Mesquita SM, Ergul E, Conta JH, Korn JM, McCarroll SA *et al*: **De novo copy number variants identify new genes and loci in isolated sporadic tetralogy of Fallot**. *Nature Genetics* 2009, **41**(8):931-935.

3. Breckpot J, Thienpont B, Peeters H, de Ravel T, Singer A, Rayyan M, Allegaert K, Vanhole C, Eyskens B, Vermeesch JR *et al*: **Array comparative genomic hybridization as a diagnostic tool for syndromic heart defects**. *J Pediatr* 2010, **156**(5):810-817.

4. Lalani SR, Shaw C, Wang X, Patel A, Patterson LW, Kolodziejska K, Szafranski P, Ou Z, Tian Q, Kang S-HL *et al*: **Rare DNA copy number variants in cardiovascular malformations with extracardiac abnormalities**. *European Journal of Human Genetics* 2012, **21**(2):173-181.

5. Soemedi R, Wilson Ian J, Bentham J, Darlay R, Töpf A, Zelenika D, Cosgrove C, Setchfield K, Thornborough C, Granados-Riveron J *et al*: **Contribution of Global Rare Copy-Number Variants to the Risk of Sporadic Congenital Heart Disease**. *The American Journal of Human Genetics* 2012, **91**(3):489-501.

6. Warburton D, Ronemus M, Kline J, Jobanputra V, Williams I, Anyane-Yeboa K, Chung W, Yu L, Wong N, Awad D *et al*: **The contribution of de novo and rare inherited copy number changes to congenital heart disease in an unselected sample of children with conotruncal defects or hypoplastic left heart disease**. *Human Genetics* 2013, **133**(1):11-27.

7. Fahed A, Gelb B, Seidman J, Seidman C: **Genetics of Congenital Heart Disease: The Glass Half Empty**. Circ Res 2013, **112**(4):707-720.

8. Pierpont ME, Basson CT, Benson DW, Gelb BD, Giglia TM, Goldmuntz E, McGee G, Sable CA, Srivastava D, Webb CL: **Genetic Basis for Congenital Heart Defects: Current Knowledge: A Scientific Statement From the American Heart Association Congenital Cardiac Defects Committee, Council on Cardiovascular Disease in the Young: Endorsed by the American Academy of Pediatrics**. Circulation 2007, **115**(23):3015-3038.
